# Lignin-Derived N,S-Co-Doped Carbon Dots Enable Improved Mn_2_O_3_ Cathodes for Aqueous Zinc-Ion Batteries

**DOI:** 10.3390/nano16100581

**Published:** 2026-05-09

**Authors:** Jiahong Wang, Wenxuan Wang, Yimin Shi, Tai Peng, Daxin Liang

**Affiliations:** 1 Key Laboratory of Bio-based Material Science and Technology, Ministry of Education, Northeast Forestry University, Harbin 150040, China; wangjiahong@nefu.edu.cn (J.W.); wxwang@nefu.edu.cn (W.W.); 2 Zhengzhou Advanced Research Institute of Harbin Institute of Technology, Harbin 150001, China; 25b925071@stu.hit.edu.cn; 3 School of Materials Science and Engineering, Jiamusi University, Jiamusi 154007, China

**Keywords:** lignin-based carbon dots, Mn_2_O_3_, aqueous zinc-ion batteries, electrochemical performance

## Abstract

Aqueous zinc-ion batteries (AZIBs) are highly promising for large-scale energy storage applications owing to their distinct merits, such as exceptional safety, abundant zinc reserves, high ionic conductivity, and facile manufacturing. Featuring natural abundance, low cost, environmental benignity, and high theoretical specific capacity, Mn_2_O_3_ has emerged as one of the most competitive cathode candidates for AZIBs. However, the low electrical conductivity of Mn_2_O_3_ impedes electron transport within the electrode, leading to significant polarization during charging and discharging and poor rate performance. Therefore, this study focuses on Mn_2_O_3_, and combines it with lignin-derived N,S-co-doped carbon dots (NS-CDs). Through a composite modification strategy, efficient conductive pathways are constructed and the structure of Mn_2_O_3_ is stabilized simultaneously, thereby effectively enhancing the electrical conductivity of the modified cathode. The incorporation of NS-CDs improves the high-rate response of the Mn_2_O_3_ cathode, with the optimized composite retaining capacity stability at 5 A g^−1^. At 0.2 A g^−1^, the specific capacity reaches 174 mAh g^−1^, and at a current density of 1 A g^−1^, the material can sustain 1000 cycles. These results highlight biomass-derived carbon dots as a viable interfacial modifier for Mn-based AZIB cathodes.

## 1. Introduction

In recent years, the continuous depletion of fossil fuels and the rapid growth in global energy storage demand have drawn widespread attention to electrochemical energy storage systems [1]. Currently, lithium-ion batteries (LIBs) continue to dominate the commercial market [2]. However, LIBs still suffer from drawbacks such as high costs, poor safety, and scarcity of lithium resources, which significantly limit their application and development across various fields. To address these issues, researchers have been actively seeking alternatives [3,4]. Aqueous zinc-ion batteries (AZIBs) have seen rapid development in recent years due to their advantages [5], including abundant zinc resources, a low redox potential (−0.76 V vs. SHE), high safety, large specific capacity, and the inherent safety of aqueous electrolytes [6,7,8].

Currently, reported cathode materials primarily include Mn-based oxides [9], V-based oxides [10], Prussian blue [11], and others. Among the numerous cathode materials, manganese-based oxides have attracted considerable attention due to their multivalent nature and high theoretical capacity [12]. Mn_2_O_3,_ as a typical trivalent manganese oxide, possesses a unique crystal structure and a high theoretical specific capacity, and has gradually emerged as one of the key research focuses for AZIB cathode materials in recent years [13]. However, Mn_2_O_3_ cathodes still face significant challenges in practical applications. Firstly, their poor electronic conductivity and the slow diffusion kinetics of zinc ions limit their rate capability and capacity. Moreover, structural distortion caused by the Jahn–Teller effect and the dissociative dissolution of manganese during cycling lead to rapid capacity decay [14,15,16,17].

Various strategies are typically employed, such as heteroatom doping, material composites and morphological control [18,19,20]. These approaches are employed to address the inherent defects of Mn_2_O_3_ cathodes. Among these, compositing Mn_2_O_3_ cathodes with carbon materials is the most effective method for enhancing their conductivity. Common carbon materials include graphene [21], carbon nanotubes [22] and others. Through physical compositing or in situ compositing, these carbon materials are uniformly coated onto or intercalated within the Mn_2_O_3_ matrix, thereby constructing a three-dimensional, continuous and highly efficient electronic transport network [23,24]. This structure significantly shortens the electron transport path, markedly reducing both the internal electron transport impedance and interfacial contact resistance within the electrode material, thereby effectively mitigating the electrochemical polarization issues caused by slow electron transport during charge–discharge cycling [25,26]. Wang et al. [27] prepared a novel, highly reversible Mn-based microsphere cathode material (Mn_2_O_3_/C) using a metal–organic framework template. The carbon component improves electronic transport and is associated with more stable cycling behavior. Fan et al. [28] constructed a unique two-dimensional (2D) layered structure of Mn_2_O_3_ and graphene. Mn_2_O_3_ nanosheets were grown perpendicularly on several layers of graphene via a rapid one-step molten salt method. Owing to the unique 2D layered structure, which possesses high electronic and ionic conductivity, as well as the synergistic effects between Mn_2_O_3_ and graphene, this cathode exhibits outstanding electrochemical performance in AZIBs. Lignin-derived carbon dots (CDs), as a novel type of nanocarbon material, not only possess the inherent advantages of traditional CDs, but also owing to the natural aromatic structure and unique functional group characteristics of lignin, offer distinct advantages such as a simple preparation process, low cost, good biocompatibility and environmental friendliness [29,30,31]. Furthermore, the types and quantities of surface functional groups can be flexibly controlled through modification processes, thereby further broadening their scope of application [32,33]. The surface of CDs typically bears functional groups such as hydroxyl and carboxyl groups; certain preparation methods can introduce amino groups and sulphur elements, thereby further enhancing their reactivity and functional properties, offering a novel approach to improving the cathode performance of AZIBs [34].

In this study, alkali lignin was used as the carbon source to synthesize N-S co-doped lignin-based carbon dots (NS-CDs) via a hydrothermal synthesis method. By leveraging the advantages of NS-CDs—namely their high conductivity, large specific surface area and strong structural modifiability—highly efficient conductive pathways were directly established, simultaneously stabilizing the structure of the material. Furthermore, by modifying the Mn_2_O_3_ cathode, the electrical conductivity of Mn_2_O_3_ as a cathode material is enhanced, thereby improving its specific capacity and cycling stability. The NS-CDs-modified Mn_2_O_3_ cathode exhibits a significantly improved specific capacity (174 mAh g^−1^) at a current density of 0.2 A g^−1^. It also demonstrated a cycle life of 1000 cycles in long-term cycling tests at 1 A g^−1^ and exhibited excellent rate capability at higher current densities (5 A g^−1^). Its overall electrochemical performance was markedly superior to that of Mn_2_O_3_, fully demonstrating the advantages of carbon material doping and modification in enhancing the performance of AZIB cathodes.

## 2. Materials and Methods

### 2.1. Synthesis of Lignin-Based CDs/Mn_2_O_3_

NS-CDs were prepared using alkali lignin as the raw material via a bottom-up hydrothermal method. Weigh 1.0 g of alkali lignin and mix it with 40 mL of H_2_O, stirring for 10 min. Then, slowly add 4.5 mL of hydrogen peroxide dropwise while continuing to stir for another 10 min. Next, add 0.03 g of thiourea to the system as a dopant that simultaneously provides N and S sources. Place the beaker in a light-protected environment and stir continuously for 8 h. Then, transfer the precursor mixture to a PTFE-lined hydrothermal reactor and place it in an oven for hydrothermal reaction at 180 °C for 12 h. Upon completion of the hydrothermal reaction, the mixture was preliminarily separated by vacuum filtration, yielding a clear, transparent filtrate. The filtrate was transferred to a dialysis bag and dialyzed for three days using deionized water as the dialysis medium.

A total of 0.98 g of Mn(CH_3_COO)_2_·4H_2_O and 0.48 g of urea were added to deionized water. The mixed solution was placed in a hydrothermal reactor and reacted at 180 °C for 12 h to obtain the precursor MnCO_3_. Finally, the precursor powder was placed in a muffle furnace and calcined in air at 450 °C for 2 h to obtain the product Mn_2_O_3_. A total of 0.01 g, 0.02 g, and 0.03 g of NS-CDs were weighed and mixed with 1.0 g of Mn_2_O_3_ powder. The reaction was carried out at 140 °C for 3 h to obtain the NS-CDs/Mn_2_O_3_ (MC) composite material. Depending on the different contents of NS-CDs in the reaction, the samples were labeled as MC-1, MC-2, and MC-3.

### 2.2. Material Characterization

The microstructure of the MC samples was characterized by scanning electron microscopy (SEM) (ZEISS-Sigma 300, Carl Zeiss AG, Oberkochen, Germany). By combining SEM with energy dispersive spectroscopy (EDS), the elemental distribution was qualitatively and quantitatively studied. The lattice fringes of the samples were analyzed using transmission electron microscopy (TEM) (Talos F200X G2, Thermo Fisher Scientific, Waltham, MA, USA) and its high-resolution (HRTEM) mode. The phase composition and crystal structure of the samples were analyzed by X-ray diffraction (XRD) (X’Pert 3 Power, Malvern Panalytical, Almelo, The Netherlands). The diffraction angle range was 10–80°. In addition, the valence state of surface elements in the MC samples was studied by X-ray photoelectron spectroscopy (XPS), and the elemental content was determined. Raman spectroscopy (inVia, Renishaw, Gloucestershire, UK) and Fourier-transform infrared spectroscopy (Nicolet 6700, Thermo Fisher Scientific, Waltham, MA, USA) were used to analyze the molecular structure. Determine the carbon content of the material using a thermal gravimetric analyzer (TGA) (NETZSCH STA 449 F5, NETZSCH, Selb, Bavaria, Germany).

### 2.3. Electrochemical Measurement

First, mix the prepared material with Super-P and PVDF in a ratio of 7:2:1 in a mortar and thoroughly and evenly grind them. Then, add an appropriate amount of N-methylpyrrolidone (NMP) as a solvent and continue wet grinding until a uniform viscosity and no particle agglomeration are achieved in the resulting mixed slurry. Apply the obtained slurry evenly onto the surface of the stainless steel foil current collector, and place it in a vacuum drying oven at 70 °C for 12 h to completely remove the solvent NMP and ensure that the binder fully forms a film. Cut the dried film into circular pieces with a diameter of 12 mm, and the average loading of the active substance on the electrode sheet is approximately 1.2 mg cm^−2^. The electrolyte consisted of 2 M ZnSO_4_ and 0.1 M MnSO_4_. The material was assembled into a button cell (CR2025) to investigate its electrochemical performance. Cyclic voltammetry (CV) and electrochemical impedance spectroscopy (EIS) tests were conducted using an electrochemical workstation (CS2350H, CorrTest, Wuhan, China). Constant-current charge–discharge performance and rate capability were evaluated using the LAND battery testing system (LAND, LANHE, Wuhan, China). Galvanostatic intermittent titration technique (GITT) was also performed.

## 3. Results and Discussion

### 3.1. Structural Evolution and Chemical Modulation of NS-CDs/Mn_2_O_3_ (MC)

**Figure 1:**
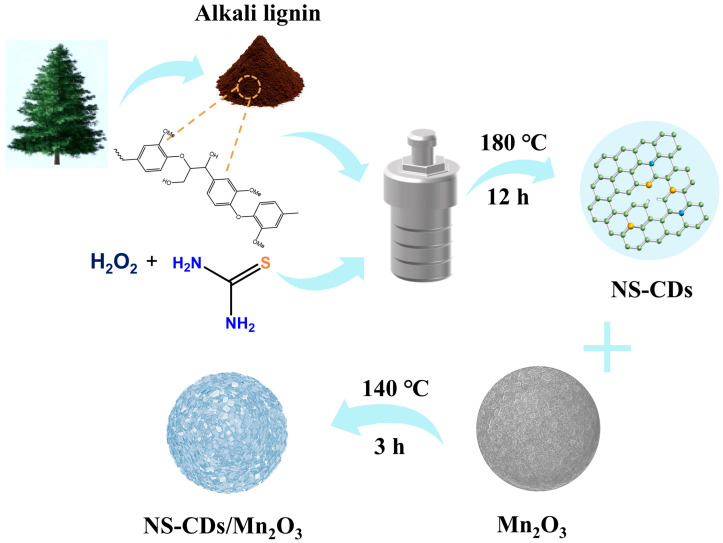
Schematic illustration of the NS-CDs/Mn_2_O_3_ synthesis process.

The synthesis steps for MC are outlined as follows in Figure 1. The first step involves hydrothermal carbonization of alkali lignin. Under high-temperature, high-pressure hydrothermal conditions (180 °C, 1.2 MPa), this process causes the alkali lignin molecules to undergo dehydration, decarboxylation, molecular framework rearrangement, aromatization, cross-linking polymerization, and gradual carbonization, ultimately forming a carbon core with a graphite-like structure; concurrently, N and S heteroatoms in the system are in situ doped into the carbon core structure or enriched on the surface of carbon sites, providing excellent performance for subsequent applications. The hydrothermally treated sample is then purified to obtain the desired NS-CDs. The second step involves hydrothermally compositing the NS-CDs with Mn_2_O_3_ to produce the MC cathode material.

**Figure 2:**
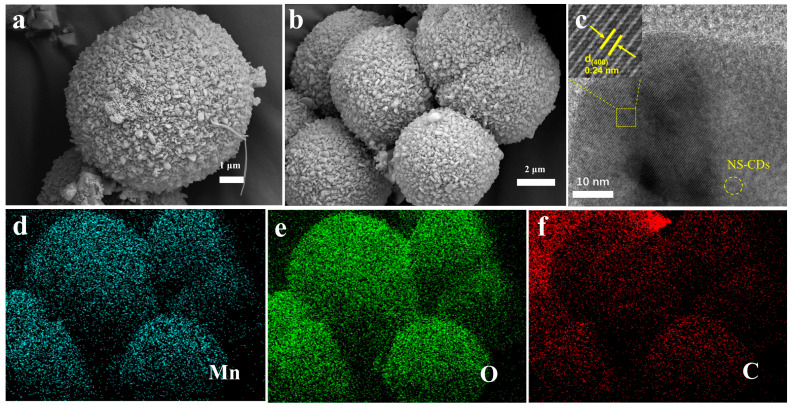
(**a**,**b**) are the SEM images of MC-2 at different magnification levels. (**c**) HRTEM image of MC-2. (**d**–**f**) SEM elemental mapping images of MC-2.

Figure S1 shows that the particle size of the NS-CDs ranges from 2 to 5 nm, with the particles generally exhibiting a regular, spherical morphology. Figure S2 shows its XRD pattern. Furthermore, distinct lattice striations can be observed in the high-resolution transmission electron microscope images, indicating that the NS-CDs possess a certain degree of graphitization and are not entirely amorphous. Figure 2a,b show the SEM images of MC-2 at different magnifications. It can be observed that the material as a whole presents a uniform micron-scale spherical morphology, with a radius of approximately 2 μm. Each microsphere is not a dense entity but is composed of a large number of nanoscale particles closely packed together, presenting a rough and porous wrinkled texture on the surface. This structure can provide rich channels for the transmission of electrolyte ions and simultaneously increase the contact area between the electrode and the electrolyte. Compared with unmodified Mn_2_O_3_ (Figure S3), the Mn_2_O_3_ microspheres subjected to hydrothermal treatment in the presence of NS-CDs exhibit a marked reduction in particle size. This size reduction arises from the multifunctional interfacial interactions between NS-CDs and Mn_2_O_3_, which suggests that the CDs influence the surface growth or aggregation behavior of Mn_2_O_3_ during post-synthesis processing. Clear lattice fringes can be observed in the HRTEM of Figure 2c, with a crystal plane spacing of 0.24 nm, corresponding to the (400) crystal plane of Mn_2_O_3_, indicating good crystallinity of the material. Meanwhile, NS-CDs with a size of approximately 2–5 nm can be observed inside Mn_2_O_3_. The above results confirm that NS-CDs have been successfully incorporated into Mn_2_O_3_. EDS energy spectrum was used for elemental analysis of MC-2. As shown in Figure 2d,f, the sample was mainly composed of three elements, Mn, O and C, and no obvious impurity elements were detected, and the three elements showed uniform co-distribution characteristics on microspheres.

**Figure 3:**
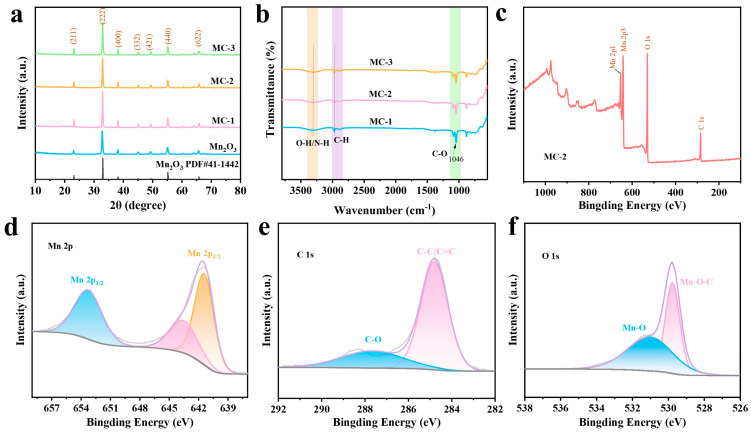
(**a**) XRD patterns of MC and Mn_2_O_3_. (**b**) FTIR spectra of MC. (**c**–**f**) XPS spectra of MC-2.

Figure 3a illustrates the effect of NS-CDs doping on the crystal structure of Mn_2_O_3_. Within the scanning range tested, the positions of the diffraction peaks for all samples remained highly consistent (corresponding to standard card PDF#41-1442), with no apparent peak shifts or the emergence of new diffraction peaks. This phenomenon indicates that the introduction of NS-CDs did not alter the intrinsic crystal structure of the Mn_2_O_3_ matrix, the material as a whole retained the crystalline structure of Mn_2_O_3_, and that the NS-CDs doping strategy exhibits good structural compatibility. From the FTIR graph in Figure 3b, it can be seen that the MC composite material was successfully prepared. In the spectrum, all samples showed broad and strong absorption peaks in the 3500–3000 cm^−1^ wavenumber range, which belong to the stretching vibrations of O-H and N-H bonds. In the 1300–800 cm^−1^ range, all samples showed characteristic absorption peaks at 1046 cm^−1^, corresponding to the stretching vibration of C-O bonds on the CDs. This is a typical structural feature of lignin-derived CDs, directly confirming that NS-CDs have been successfully introduced into the Mn_2_O_3_ matrix. The characteristic absorption of Mn-O bonds in Mn_2_O_3_ mainly occurs in the low wavenumber region below 800 cm^−1^, and is covered by the absorption peaks of NS-CDs. The FTIR spectra of the NS-CDs are shown in Figure S4. Furthermore, based on TG analysis, the content of NS-CDs in the MC material can be determined (Figure S5).

The surface elemental composition, chemical valence states and interfacial interactions of MC-2 were characterized using XPS. Figure 3c shows the full XPS spectrum of MC-2, which clearly reveals characteristic peaks for Mn 2p, O 1s and C 1s; no other impurity elements were detected. Figure 3d shows the high-resolution Mn 2p spectrum, which consists of two spin–orbit split peaks, Mn 2p_3/2_ and Mn 2p_1/2_. Mn is primarily present in the Mn_2_O_3_ lattice in the +3 oxidation state; simultaneously, some Mn ions form coordination bonds with oxygen-containing functional groups on the NS-CDs surface, resulting in a slight shift in the binding energy. Figure 3e shows the C 1s fine-structured spectrum, revealing two characteristic peaks: C-C/C=C (284.8 eV) and C-O (286.3 eV). The C–C/C=C peak corresponds to the sp2-hybridized graphitized carbon framework of the NS-CDs, which is a typical feature of carbon materials and confers good electrical conductivity on the composite; the C-O peak, on the other hand, originates from the oxygen-containing functional groups on the surface of the NS-CDs. Figure 3f shows the high-resolution O 1s spectrum, which exhibits two characteristic peaks: Mn-O (531.8 eV) and Mn-O-C (530.9 eV). The Mn-O peak is attributed to the Mn-O bond within the Mn_2_O_3_ lattice and represents an intrinsic signal of the matrix. XPS analysis comprehensively confirms the successful composite formation and interfacial chemical interactions between NS-CDs and Mn_2_O_3_; the graphitized carbon framework provides an electronic conductive network for the composite material, enabling it to exhibit excellent electrochemical performance in AZIBs.

### 3.2. Electrochemical Performance of the MC Cathode of AZIBs

**Figure 4:**
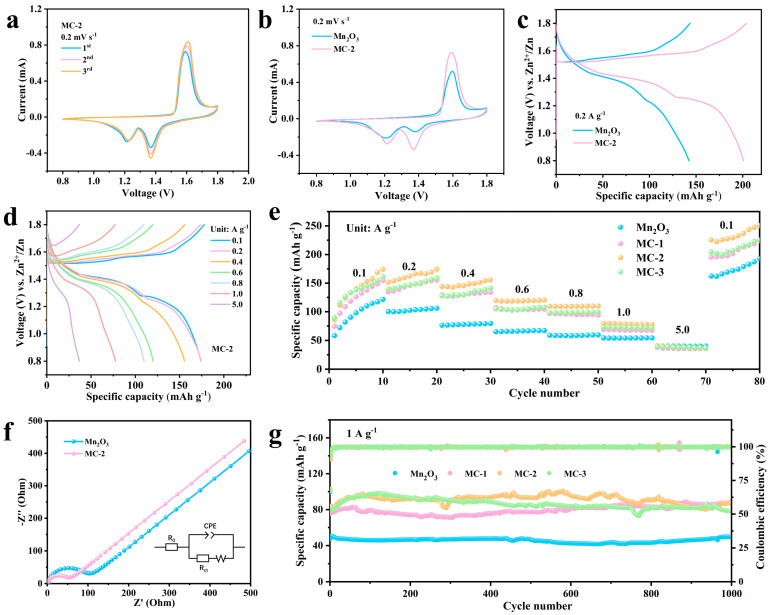
(**a**) CV curve of MC-2 at 0.2 mV s^−1^. (**b**) Comparison of the CV curves of MC-2 and Mn_2_O_3_ at 0.2 mV s^−1^. (**c**) Comparison of CGD curves for MC-2 and Mn_2_O_3_ at 0.2 A g^−1^. (**d**) GCD curves of the MC-2 at different current densities. (**e**) Rate performance of the materials at various current densities. (**f**) Plot of EIS fitting curves for MC-2 and Mn_2_O_3_. (**g**) Cycle performances of the materials.

To thoroughly evaluate the electrochemical redox behavior and structural stability of the MC cathode material in AZIBs, CV tests were conducted at a scan rate of 0.2 mV s^−1^. Figure S6 shows the CV curves of MC-2 in different electrolytes. In comparison, Figure 4a and Figure S7 show the first three cycles of CV curves for MC cathodes with different NS-CDs doping levels at a scan rate of 0.2 mV s^−1^. All samples exhibited typical redox peaks, with two reduction peaks appearing in the 1.2–1.5 V range, corresponding to the process of Zn^2+^ insertion into the cathode material; an oxidation peak appears at approximately 1.6 V, corresponding to the process of Zn^2+^ desorption from the cathode material. The curves from the first to the third cycles are essentially superimposed, with no significant shift in the positions or shapes of the oxidation/reduction peaks, and only minor changes in peak current, which indicates that all three groups of samples exhibit good electrochemical reversibility and structural stability during the initial cycles, with no significant side reactions or irreversible structural collapse occurring, as shown in Figure 4b, which compares the CV curves of MC in the first cycle. Under the same test conditions, the active material loading of Mn_2_O_3_ and MC-2 is 1.215 mg cm^−2^ and 1.19 mg cm^−2^, respectively, and the area enclosed by the CV curve of MC-2 is larger than that of Mn_2_O_3_. This indicates that MC-2 has higher electrochemical performance [13].

Galvanostatic charge–discharge (GCD) was conducted on the material at a current density of 0.2 A g^−1^. As clearly shown by the GCD curves (Figure 4c), compared with pristine Mn_2_O_3_, MC-2 shows a more defined voltage plateau and higher reversible capacity, consistent with reduced polarization after NS-CD incorporation. Figure 4d and Figure S8 show the GCD curves of MC at different current densities; all curves exhibit two distinct voltage plateaus, which are closely related to the redox reactions of the Mn element in the material and the energy storage mechanism involving the co-insertion and co-extraction of Zn^2+^ and H^+^ [35]. At a low current density, the longer charge/discharge time allows more complete ion insertion and extraction, leading to clearer voltage plateaus and higher reversible capacity, allowing Zn^2+^ and H^+^ to be fully intercalated into and deintercalated from the Mn_2_O_3_ lattice, resulting in good reaction reversibility. As the current density was gradually increased (0.2, 0.5, 1.0, 5.0 A g^−1^), the GCD curves of all samples exhibited a trend of shortened plateaus, increased voltage differences and decreased specific capacity. The reason for this is that at high current densities, the rates of ion insertion and desorption accelerate, and lattice diffusion becomes the rate-limiting step. Consequently, some Zn^2+^ and H^+^ are unable to complete insertion or desorption in time, leading to intensified polarization. At the same time, insulating zinc products are prone to form on the surface of the active material, impeding ion transport and further reducing the specific capacity output. A comparison reveals that MC-2 exhibits the best discharge specific capacity. At current densities of 0.1, 0.2, 0.4, 0.6, 0.8, 1.0 and 5.0, the discharge specific capacity of MC-2 was 174.2, 174, 155.6, 120.2, 109.9, 77.5 and 37 mAh g^−1^, respectively. As can be seen from the rate performance curve (Figure 4e), under high current densities of 5.0 A g^−1^, MC maintains a considerable discharge capacity, demonstrating excellent high-rate charge–discharge capability.

As shown in Figure 4f, which investigates the effect of NS-CD doping on the impedance of Mn_2_O_3_ electrodes, EIS testing of the MC-2 sample revealed typical Nyquist plot characteristics. The semicircular diameter of the MC-2 sample (Rct = 45.07 Ω) is smaller than that of Mn_2_O_3_ (Rct = 87.61 Ω), indicating that its interfacial charge transfer resistance is significantly lower than that of Mn_2_O_3_ (Table S1). This is attributed to the highly efficient electron transport network formed by the appropriate doping of NS-CDs, which effectively reduces interfacial impedance and accelerates electron transfer between the electrode material and the electrolyte. Furthermore, long-term cycling performance tests were conducted on the samples at a current density of 1 A g^−1^. As shown in Figure 4g, Mn_2_O_3_ exhibited the lowest specific capacity, whilst the specific capacities of the MC electrodes were all superior to that of Mn_2_O_3_. Compared with other ZIB cathode materials (Table S2), over a cycle range of 1000 cycles, the MC-2 sample demonstrated the best cycling stability and capacity retention, maintaining a capacity of 86.9 mAh g^−1^ even after 1000 cycles, with a coulombic efficiency of 99.82%, demonstrating exceptional structural stability.

**Figure 5:**
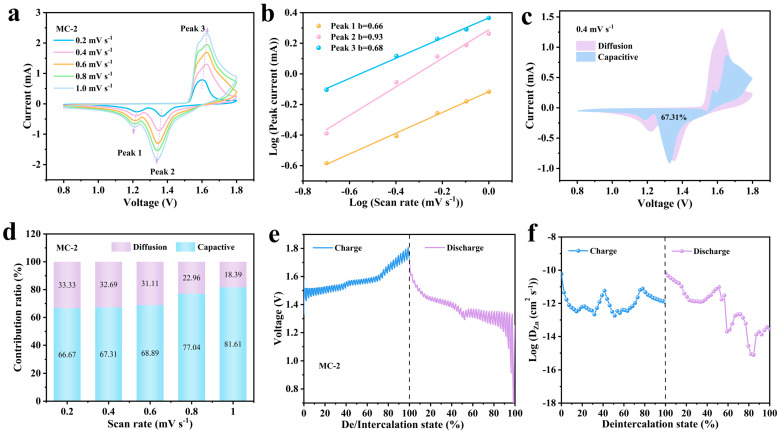
(**a**) CV curves for the MC-2 at different scan rates. (**b**) Fitted b-value. (**c**) Capacitive contribution of MC-2 at 0.4 mV s^−1^. (**d**) The ratio of capacitive contribution of MC-2. (**e**) The GITT curve for the MC-2. (**f**) Ion diffusion coefficient of MC-2.

The MC-2 electrode exhibits remarkable electrochemical performance, indicating that it possesses excellent kinetic characteristics. Therefore, the electrochemical kinetics and charge storage mechanisms of the MC-2 electrode were investigated in detail. As shown in Figure 5a, CV tests were conducted at scan rates ranging from 0.2 to 1.0 mV s^−1^ within a potential window of 0.8-1.8 V. Three distinct redox peaks can be clearly observed from the curve morphology, Peak 1 and Peak 2 are reduction peaks, contributing to the insertion of Zn^2+^ and H^+^, while Peak 3 is an oxidation peak, arising from the desorption of Zn^2+^ and the conversion between Mn^2+^ and Mn^3+^ [13]. As the scan rate increases, the peak shapes gradually broaden, the positions of the redox peaks shift, and the peak spacing increases, which is a typical example of how the electrochemical activity of an electrode varies with the scanning rate. Combining the relationship between peak current (*i*) and scan rate (*v*), quantitative analysis of surface capacitance and diffusion processes was performed using Formula (1):

*i* = *av^b^* (1)

where a and b are constants. The b-value is a crucial parameter characterizing battery kinetic performance [36]. When b equals 0.5, the electrode reaction is entirely diffusion-controlled. When b equals 1.0, the electrode reaction is entirely surface capacitance-controlled. The corresponding b values obtained from the fitting calculations (Figure 5b) are 0.66, 0.93 and 0.68, respectively, falling within the range of 0.5 to 1.0, indicating that the MC-2 electrode exhibits a hybrid mechanism involving both diffusion-controlled and capacitive-controlled processes. To further investigate the contribution ratios of diffusion control and capacitive behavior, the capacitive contribution ratio was calculated at different scan rates. At a given scan rate, it follows Formula (2).

*i* = *k*_1_*v* + *k*_2_*v*^1/2^ (2)

where *i* represents the current at each scan rate, and *k*_1_*v* and *k*_2_*v*^1/2^ denote the contributions from capacitive and diffusion control, respectively [37]. As can be seen from Figure 5c,d, at a scan rate of 0.2 mV s^−1^, the capacitive contribution was approximately 66.67%, indicating that charge storage was predominantly governed by capacitive processes. As the scan rate increased, the capacitive contribution rose to 67.31%, 68.89%, 77.04% and 81.61%, respectively.

The bulk and interfacial Zn^2+^ diffusion coefficient within the cathode material is a pivotal factor dictating the rate performance, cycling stability, and charge/discharge kinetics of batteries [38,39]. The ion diffusion coefficient (D) quantifies the migration rate of ions within an electrode material. A higher D value signifies faster Zn^2+^ transport through the bulk and interfaces [40]. To investigate the ion transport kinetics of the MC-2 electrode during charging and discharging in greater depth, this study employed GITT for characterization. Figure 5e shows the voltage profile corresponding to Zn^2+^ deintercalation/intercalation of the MC-2 electrode upon charging. During charging, the voltage rises gradually from an initial 1.45 V to 1.8 V, showing an overall gentle upward trend, corresponding to the reaction process of Zn^2+^ deintercalation from the cathode lattice. No significant voltage spikes were observed, indicating that the material exhibits good structural stability and reaction reversibility during the deintercalation stage. During the discharge stage, the voltage drops rapidly from 1.8 V to 1.3 V before entering a stable plateau region, eventually decreasing slowly to around 0.8 V during deep discharge. This voltage plateau corresponds to the main reaction stage of Zn^2+^ insertion into the cathode lattice; the prolonged duration of the plateau and minimal voltage fluctuations further validate the excellent electrochemical reversibility of the MC-2 material. The Zn^2+^ diffusion coefficient D, calculated from GITT data (Figure 5f) is generally lower during the discharge stage (D values ranging from 10^−15^ to 10^−10^ cm^2^ s^−1^) compared to the charging stage (D values ranging from 10^−13^ to 10^−10^ cm^2^ s^−1^), indicating that the diffusion resistance associated with Zn^2+^ insertion into the lattice is greater than that of the deintercalation process. As the depth of zinc insertion increases, the diffusion coefficient exhibits a decreasing trend. This is primarily attributed to the narrowing of ionic channels caused by lattice compression, as well as the enhanced electrostatic repulsion between Zn^2+^.

## 4. Conclusions

This study demonstrates that the strategy of modifying Mn_2_O_3_ with NS-CDs is an efficient, mild and environmentally friendly method for modifying cathode materials, offering significant advantages in enhancing the material’s electrical conductivity. A stable interfacial bond can be formed between NS-CDs and Mn_2_O_3_, which not only effectively suppresses the volume expansion and lattice distortion of Mn_2_O_3_ during charge–discharge cycles, thereby maintaining the integrity of its structure, but also utilizes the excellent electronic conductivity of NS-CDs to construct a continuous and efficient electronic transport network within the Mn_2_O_3_ cathode. Concurrently, it widens the diffusion pathways for Zn^2+^ in the electrolyte, accelerating the ion migration rate. At a current density of 0.2 A g^−1^, the specific capacity of the MC can reach 174 mAh g^−1^, and at a current density of 1 A g^−1^, it can sustain 1000 cycles. Furthermore, it exhibits excellent rate performance at high current densities, maintaining stable capacity even at 5 A g^−1^. Taken together, the data show that NS-CD incorporation is an effective way to improve the electrochemical response of Mn_2_O_3_ under the present testing conditions, further enhancing its specific capacity, rate capability and long-term cycling stability, thereby providing a novel approach and methodology for the development of high-performance AZIB cathode materials.

## Data Availability

The original contributions presented in this study are included in the article/Supplementary Materials. Further inquiries can be directed to the corresponding authors.

## Supplementary Materials

The following supporting information can be downloaded at https://www.mdpi.com/article/10.3390/nano16100581/s1, Figure S1: TEM images of NS-CDs; Figure S2: XRD patterns of NS-CDs; Figure S3: SEM images of different samples: (a) Mn_2_O_3_. (b) MC-1. (c) MC-3; Figure S4: FTIR spectrum of NS-CDs; Figure S5: The TG curves for MC; Figure S6: CV curves of MC-2 under different electrolyte conditions; Figure S7: CV curves of MC at 0.2 mV s^−1^: (a) MC-1, (b) MC-3; Figure S8: GCD curves of MC: (a) MC-1, (b) MC-3; Table S1: The impedance fitting values of Mn_2_O_3_ and MC-2; Table S2: Performance comparison between Mn_2_O_3_ materials in previously reports as cathodes for ZIBs.

## Abbreviations

The following abbreviations are used in this manuscript:

AZIBs	Aqueous Zinc-Ion Batteries
LIBs	Lithium-Ion Batteries
NS-CDs	Lignin-derived carbon dots
MC	NS-CDs/Mn_2_O_3_
SEM	Scanning Electron Microscope
TEM	Transmission Electron Microscope,
XRD	X-ray Diffraction
FTIR	Fourier-Transform Infrared Spectroscopy
TG	Thermal Gravimetric
CV	Cyclic Voltammetry
GCD	Galvanostatic Charge/Discharge
EIS	Electrochemical Impedance Spectroscopy
GITT	Galvanostatic Intermittent Titration Technique
