# Supplementary material for: Lignin-Derived N,S-Co-Doped Carbon Dots Enable Improved Mn2O3 Cathodes for Aqueous Zinc-Ion Batteries
Source: Nanomaterials (Basel). 2026 May 9;16(10):581. doi: 10.3390/nano16100581 (PMC13209756; doi:10.3390/nano16100581)
Supplement: Supplementary file 1 [file nanomaterials-16-00581-s001.zip › nanomaterials-4283765-supplementary.pdf]

Lignin-Derived Carbon Dots-Modified Mn_2O_3 as High-Performance Cathode Materials for Aqueous Zinc-Ion Batteries

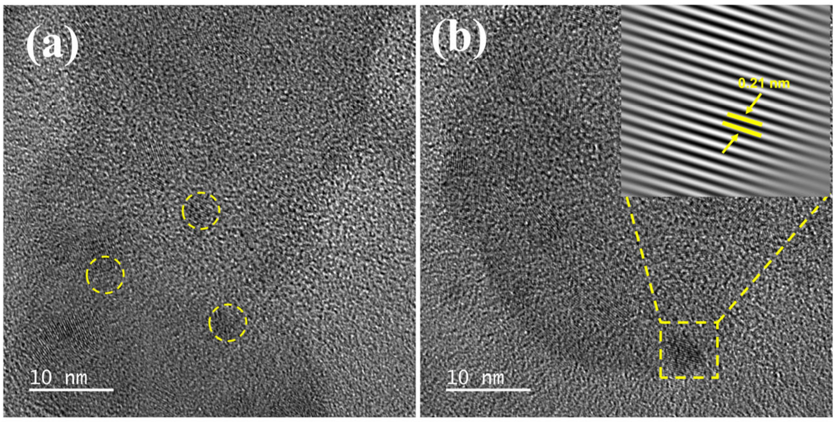

Figure S1 TEM images of NS-CDs.

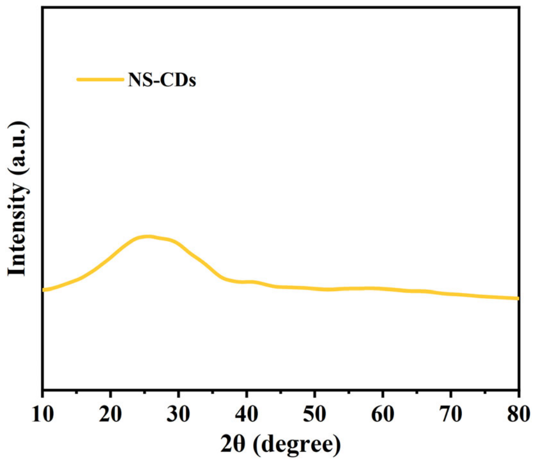

Figure S2 XRD patterns of NS-CDs.

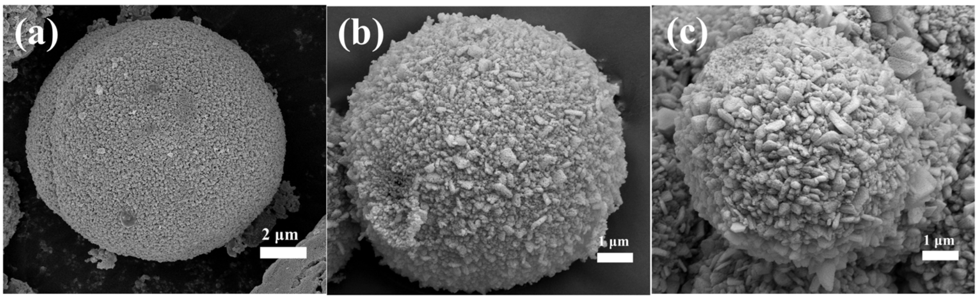

Figure S3. SEM images of different samples: (a) Mn_2O_3. (b) MC-1. (c) MC-3.

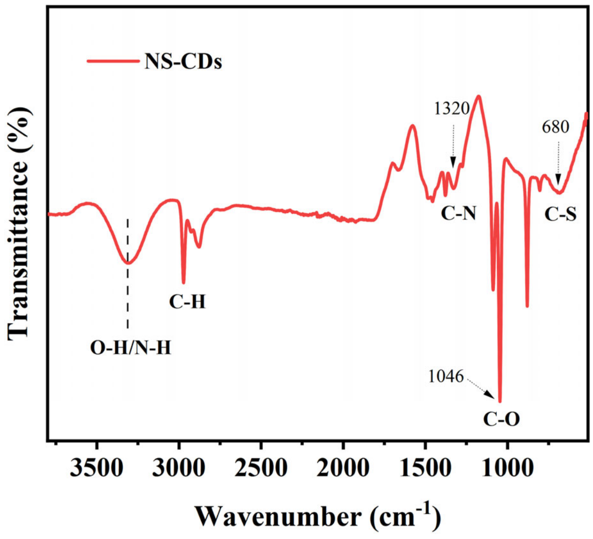

Figure S4 FTIR spectrum of NS-CDs.

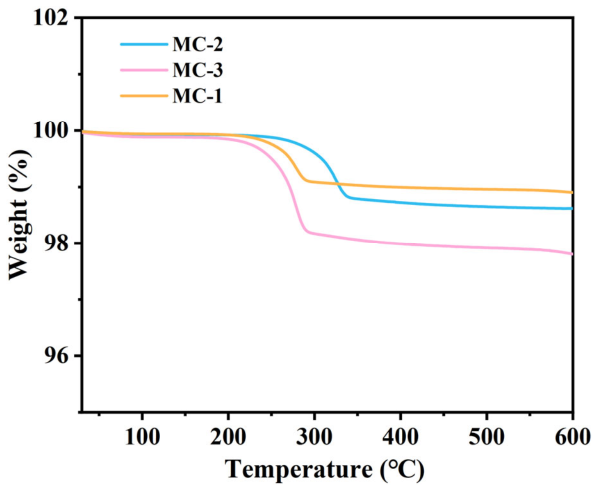

Figure S5 The TG curves for MC

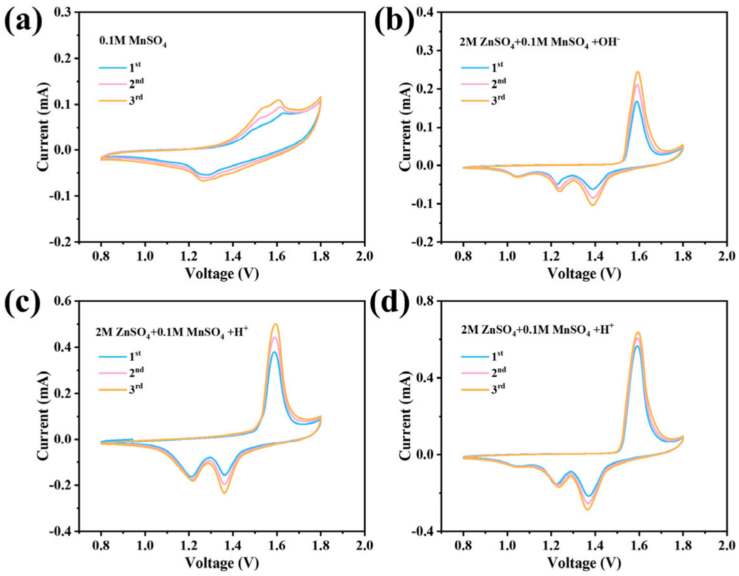

Figure S6 CV curves of MC-2 under different electrolyte conditions

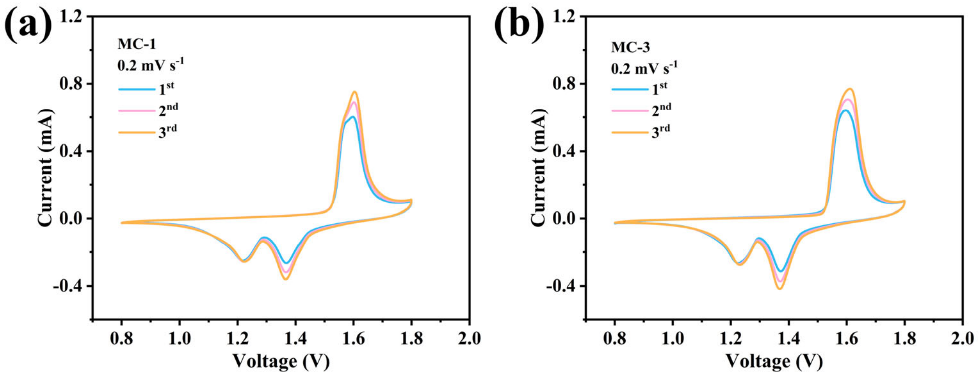

Figure S7 CV curves of MC at 0.2 mV s-1: (a) MC-1, (b) MC-3

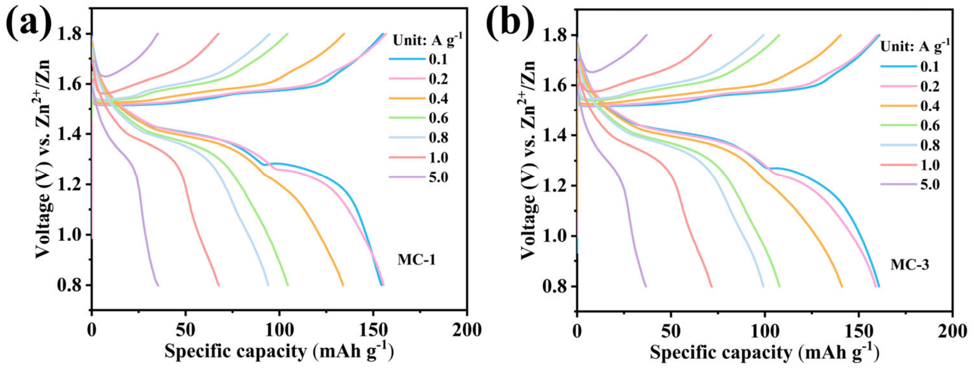

Figure S8 GCD curves of MC: (a) MC-1, (b) MC-3

Table S1 The impedance fitting values of Mn_2O_3 and MC-2

	Mn_2O_3	MC-2
Rct(Ω)	87.61	45.07

Table S2. Performance comparison between Mn_2O_3 materials in previously reports as cathodes for ZIBs.

**Materials**	**Specific capacity**	**Current density**	**Cycling performance**	**Reference**
$ZnMn_2O_4/Mn_2O_3$	109 mAh g^{-1}	0.05 C	150	[1]
Mn_2O_3 nanofiber	56 mAh g^{-1}	1 A g^{-1}	500	[2]
$Na_{0.44}MnO_2/Mn_2O_3$	68.3 mAh g^{-1}	0.4 A g^{-1}	200	[3]
Mn_2O_3@PPy	43 mAh g^{-1}	1 A g^{-1}	1000	[4]
MnO_2-Mn_2O_3	84.7 mAh g^{-1}	1 A g^{-1}	1000	[5]
Amorphous Mn_3O_4	128 mAh g^{-1}	0.1 A g^{-1}	200	[6]
Cu-Mn_3O_4	44.5 mAh g^{-1}	1 A g^{-1}	-	[7]
Mo-Mn_2O_3	63.5 mAh g^{-1}	1 A g^{-1}	1000	[8]
**NS-CDs/Mn_2O_3**	**86.9 mAh g^{-1}**	**1 A g^{-1}**	**1000**	**This Work**
